# Effects of Antihypertensive Treatment on Left and Right Ventricular Global Longitudinal Strain and Diastolic Parameters in Patients with Hypertension and Obstructive Sleep Apnea: Randomized Clinical Trial of Chlorthalidone plus Amiloride vs. Amlodipine †

**DOI:** 10.3390/jcm12113785

**Published:** 2023-05-31

**Authors:** Juliano A. Jorge, Murilo Foppa, Angela B. S. Santos, Fábio T. Cichelero, Denis Martinez, Marcelo B. Lucca, Geórgia P. F. de Oliveira, Flávio D. Fuchs, Sandra C. Fuchs

**Affiliations:** 1 Graduate Program in Cardiology, School of Medicine, Universidade Federal do Rio Grande do Sul (UFRGS), Porto Alegre 90035-903, RS, Brazil; sojuliano@hotmail.com (J.A.J.); mufoppa@hcpa.edu.br (M.F.); abssantos@hcpa.edu.br (A.B.S.S.); dr.fabio1@gmail.com (F.T.C.); denis@sono.com.br (D.M.); marceloblucca@gmail.com (M.B.L.); ffuchs@hcpa.edu.br (F.D.F.); 2 INCT PREVER, Hospital de Clínicas de Porto Alegre (HCPA), School of Medicine, Universidade Federal do Rio Grande do Sul (UFRGS), Porto Alegre 90035-903, RS, Brazil; georgiapantef@gmail.com; 3 Division of Cardiology, Hospital de Clínicas de Porto Alegre (HCPA), Porto Alegre 90035-903, RS, Brazil

**Keywords:** diastolic function, global longitudinal strain, left ventricular, randomized controlled trial, obstructive sleep apnea, hypertension, echocardiography

## Abstract

Hypertension is highly prevalent in patients with obstructive sleep apnea (OSA), and fluid retention with its nighttime rostral distribution is one potential mechanism. We tested whether or not diuretics differ from amlodipine in their impact on echocardiographic parameters. Patients with moderate OSA and hypertension were randomized to receive diuretics (chlorthalidone plus amiloride) or amlodipine daily for 8 weeks. We compared their effects on left and right ventricular global longitudinal strain (LV-GLS and RV-GLS, respectively), on LV diastolic parameters, and on LV remodeling. In the 55 participants who had echocardiographic images feasible for strain analysis, all echocardiographic parameters were within normal ranges. After 8 weeks, the 24 h blood pressure (BP) reduction values were similar, while most echocardiographic metrics were kept unchanged, except for LV-GLS and LV mass. In conclusion, the use of diuretics or amlodipine had small and similar effects on echocardiographic parameters in patients with moderate OSA and hypertension, suggesting that they do not have important effects on mediating the interaction between OSA and hypertension.

## 1. Introduction

Obstructive sleep apnea (OSA) is characterized by recurrent complete and partial upper airway obstructive events, resulting in intermittent hypoxemia, sleep fragmentation, and daytime sleepiness. It is associated with a high prevalence of hypertension, as much as it is with heart failure, ischemic heart disease, pulmonary hypertension, arrhythmia, and stroke [1].

It is known that there is a long installation period for these cardiovascular maladaptive mechanisms, and echocardiography permits the noninvasive evaluation of the cardiac structures and preclinical cardiac systolic and diastolic dysfunction [2]. More recently, speckle tracking global longitudinal strain has been shown to be more sensitive to and accurate for identifying these early changes in cardiac function in hypertensive patients [3].

The interaction between OSA and hypertension may lead to the development of cardiac systolic and diastolic dysfunction, but the underlying mechanisms are not well-understood. Dysautonomia and repetitive hypoxia induced by obstructive sleep apnea (OSA) can affect the interaction between oxygen supply and demand to the myocardium, leading to myocardial ischemia [4,5], which could impair ventricular systolic function. Body fluid accumulation and the mechanism of OSA in hypertensive patients may involve the movement of fluid from the lower limbs to the cervical region during sleep [6], and diuretic therapy improves the apnea–hypopnea index (AHI) in these patients [7,8].

Continuous positive airway pressure (CPAP) improves blood pressure control in patients with OSA and resistant hypertension [9], particularly nighttime blood pressure [10]. However, CPAP’s impact on quality of life [11], and cardiovascular event prevention [12] is less consistent according to clinical trial results. There are also well-known limitations related to adherence to CPAP use [13]. Inversely, the impact of anti-hypertensive treatment on OSA severity and its complications is less studied.

In summary, there are multiple connections between OSA, hypertension, and their cardiac dysfunctions. In this context, we tested in a randomized clinical trial the effects of a combination of chlorthalidone plus amiloride vs. amlodipine over a period of eight weeks on echocardiographic parameters of left and right ventricular function in patients with OSA and hypertension.

## 2. Methods

### 2.1. Study Design

This parallel randomized clinical trial was conducted in a university hospital and approved by the research ethics committee (GPPG: 2015-0274), which is accredited by the Office of Human Research Protection as an institutional review committee (IRB0000921). The study was performed in compliance with the amended Declaration of Helsinki. The protocol was uploaded to clinicaltrials.gov ((NCT 01896661 and NCT 02896621) and published [14]. All participants signed an informed consent form prior to participation in the main trial.

### 2.2. Study Population

Participants aged 40 years or older, who had undergone complete polysomnography (PSG) in the sleep laboratory, and had been diagnosed with OSA and hypertension were eligible. Hypertension was confirmed via 24 h ambulatory blood pressure monitoring (ABPM), with values of ≥130/80 mmHg, an office blood pressure (BP) of ≥140/90 mmHg or the use of antihypertensive medication. Individuals who were taking BP-lowering drugs were required to undergo a two-week washout period to determine their eligibility. The exclusion criteria comprised allergies or intolerance to the experimental drugs, secondary hypertension diagnosis, current treatment for OSA, recent diagnosis of myocardial infarction or stroke within three months, sustained arrhythmias, heart failure, low life expectancy, ongoing pregnancy, or participation in another study within the past six months.

### 2.3. Intervention

The participants were randomized to receive a combination of diuretics (chlorthalidone 25 mg plus amiloride 5 mg) or amlodipine 10 mg, administered for eight weeks as a single daily dose in the morning without dose titration. Treatment allocation was randomized and stratified by sex and OSA severity (AHI > 5–25 and 26–40 events/h) in blocks of varying sizes. Before patient enrollment, the randomization list was generated by an independent researcher (who had no contact with the participants) in Random Allocation Software 1.0 and was stored in the Research Electronic Data Capture platform (REDCap—Vanderbilt University; Nashville, TN, USA). The treatment groups were concealed from the participants, doctors, and researchers until the completion of the study.

### 2.4. Outcomes

Participants were evaluated at baseline with anthropometric evaluation including height, weight, and cervical circumference, laboratory tests (creatinine, potassium, fasting glucose, and NT-proBNP), transthoracic echocardiography, 24 h ABPM, and total body water via bioimpedance (Biospace InBody 230; Biospace Co., Seoul, Republic of Korea). These measures were repeated at the end of the study.

The primary endpoints were the variation after the eight weeks of left and right ventricular longitudinal strain (LV-GLS and RV-GLS, respectively), and Doppler LV diastolic parameters (lateral e′, septal e′, E/e′ ratio, and E/A ratio). The secondary outcomes were changes in 24 h ABPM measures, LV ejection fraction (LVEF), LV mass, relative wall thickness (RWT), left atrial volume index (LAVI), and left ventricular outflow tract velocity time integral (LVOT-VTI).

Adverse events were investigated through self-reported symptoms in a semi-structured questionnaire.

### 2.5. Echocardiography

All participants underwent a transthoracic echocardiographic examination (Phillips CX-50; Bothell, WA, USA) with an adult sector transducer (1–5 MHz) by the same trained certified echocardiographer (JAJ), who was blinded to participants’ medication. Two-dimensional and Doppler images were acquired according to the recommendations of the American Society of Echocardiography and the European Association of Cardiovascular Imaging (ASE/EACI) [2].

The mean of three consecutive heart beat measurements was used in the analysis. In the parasternal longitudinal view, LV diastolic and systolic diameters, septal, and posterior wall thicknesses were measured to calculate LV mass and relative wall thickness (RWT = 2 × posterior wall/LV diastolic diameter). Final LV diastolic and systolic and left atrial systolic volumes and LV ejection fraction (LVEF) were measured in 4-chamber and 2-chamber apical views using a modified Simpson’s rule. LV mass and left atrial volume were indexed to body surface area (LVMI and LAVI, respectively). Early (E-wave) and late (A-wave) peak velocities were measured from spectral traces of pulsed Doppler in mitral inflow. Tissue Doppler imaging was used to obtain early septal (septal e′) and lateral (lateral e’) mitral annulus displacement peak velocities. These parameters were used to calculate the E/A, and the mean E/e′ ratio from septal e′ and lateral e′. LVOT-VTI, a Doppler surrogate for cardiac output [15], was measured in apical views with the sample volume at the LV LVOT, just before aortic valve flow acceleration. Global longitudinal strain (GLS) was analyzed offline using previously validated and commercially available software dedicated to LV and RV analysis (2D Cardiac Performance Analysis^©^, TomTec-ArenaTM 1.2 Imaging Systems, Unterschleißheim, Germany). LV-GLS was calculated from 4- and 2-chamber apical views, and RV-GLS was calculated from a 4-chamber apical view.

### 2.6. Blood Pressure Measurement

During the study, Ambulo 2400 (Mortara, Milwaukee, WI, USA) with an adequate cuff size was employed to conduct 24 h ABPM on the non-dominant arm. Blood pressure readings were taken every 15 min while awake and every 20 min during the sleep period. Office blood pressure was measured twice using a standardized technique with an oscillometric monitor (Microlife BP 3BTO-A; Micromed, Brasília, Brazil) on two separate occasions, and the average value was calculated. The extent of nighttime BP dipping was determined as the percentage difference between the mean systolic pressure during daytime and nighttime.

### 2.7. Polysomnography

Conventional polysomnography was assessed with a BrainNet digital instrument (EMSA, Rio de Janeiro, Brazil), which recorded the electroencephalogram (EEG; C4-A1, F4-A1, O2-A1), left and right electrooculogram, and submental and tibial electromyography. Airflow was measured with a nasal cannula attached to a pressure transducer (Ultima, Braebon, Kanata, ON, Canada). Respiratory effort was evaluated using respiratory inductance plethysmography (QRIP, Braebon, Kanata, ON, Canada), while pulse oximetry (XPOD, Nonin, Plymouth, MN, USA) was employed to monitor oxygen saturation levels. Additionally, electrocardiogram readings were taken during the night in the sleep laboratory using a single derivation, DII. In this study, obstructive apnea was characterized as a reduction in inspiratory airflow by over 90% accompanied by a minimum of 10 s of respiratory effort. Hypopnea was determined by a more than 30% reduction in respiratory flow coupled with a desaturation of over 3% or an arousal. The AHI was calculated as the number of apnea and hypopnea events per hour of sleep, with more than 5 events per hour being considered abnormal.

### 2.8. Sample Size Calculation and Statistical Analysis

The sample size was estimated to be 28 participants per group, based on a difference in the E/A ratio of 0.3 ± 0.4 found in a trial of an intervention with CPAP [16], with 80% power and an alpha of 0.05 (Epidat version 3.1; Dirección Xeral de Saúde Pública, Xunta de Galícia; OPS-OMS). The sample was expanded to 33 participants per group, considering potential losses in the follow up.

The results were presented as means and standard deviations and the frequencies as percentages. For continuous variables, variations between baseline and follow-up values in each group were compared using paired *t*-tests and *t*-tests for independent samples, with *p* < 0.05 being considered statistically significant. We performed additional analysis using two-way ANOVA for treatment and time and the *p* value for interaction was described. Intra-observer variability was determined via a duplicate assessment of LV- and RV-GLS. Intra-observer reproducibility was assessed with coefficient of variation and intraclass correlation coefficient (ICC) tests. The analyses were performed in Stata 12 (StataCorp, College Station, TX, USA).

## 3. Results

The database of patients having polysomnography between December 2014 and February 2017 was screened and most were excluded for not having OSA or refused to participate. Among those eligible, 66 were randomized—33 were randomized to the diuretics group (chlorthalidone plus amiloride) and 33 to the amlodipine group. One participant in the diuretics group and three in the amlodipine group were lost upon follow up, and one participant in the amlodipine group discontinued the intervention due to ankle edema but returned for the final eight-week visit. Image quality permitted the measurement of GLS in 55 participants (27 in the diuretics group and 28 in the amlodipine group), and 1 patient did not repeat the 24 h ABPM at the end of the study. The study included mostly overweight men with an average AHI of over 29 events/h. There were no significant differences between the groups in terms of their baseline demographic and clinical characteristics (Table 1). There was no significant difference observed in the effect of drugs on 24 h ABPM (Table 2) and echocardiographic (Table 3) parameters.

At the end of the eight-week intervention period, there was a significant reduction of approximately 10 mmHg in systolic and 5 mmHg in diastolic 24 h BP, with corresponding correlates in daytime and nighttime systolic and diastolic BP (Table 2). These BP reductions were of similar magnitude between the allocated groups.

Echocardiographic structural and functional parameters were within normal ranges and did not differ between the two groups at baseline or at the end of the follow-up period (Table 3). Notably, none of the participants had elevated LV filling pressures based on the echocardiographic E/e′ ratio, and only three of them presented a baseline NT-proBNP above the normal cut-off point of 125 pg/mL (one in the diuretics group and two in the amlodipine group).

**Figure 1:**
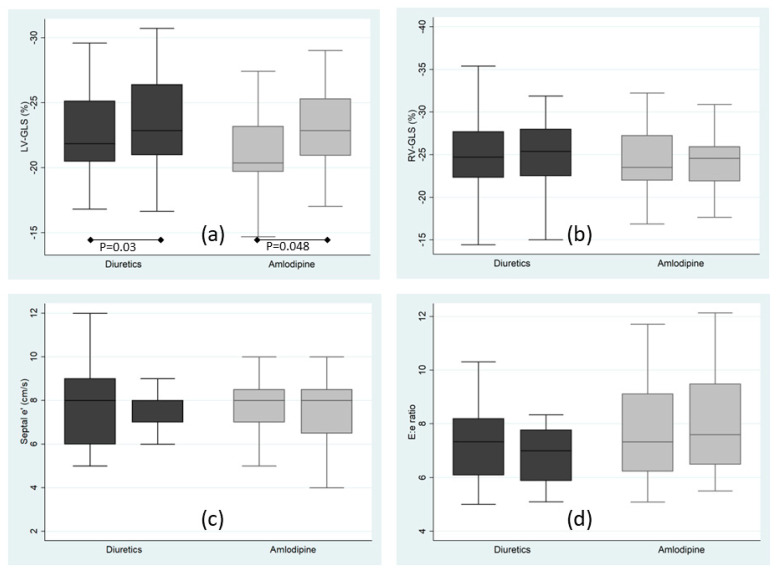
Echocardiographic primary endpoints at baseline and 8 weeks after randomization to diuretics (chlorthalidone + amiloride; dark boxes) or amlodipine (light boxes). (**a**) Left ventricular global longitudinal strain; (**b**) Right ventricular global longitudinal strain. (**c**) Tissue Doppler early septal mitral annulus displacement peak velocity (**d**) Ratio of the early mitral inflow peak velocity to the tissue Doppler early septal mitral annulus displacement peak velocity; *p* for comparison between baseline and 8 weeks measurements, the remaining comparisons were not statistically significant.

Primary outcomes are shown in Figure 1 and Table 3. Left ventricular global longitudinal strain (LV-GLS) demonstrated an improvement with no significant difference between the groups at the end of the follow-up period (*p* = 0.9). On the other hand, antihypertensive treatment had no effect on RV strain in either group. Left ventricular ejection fraction (LV-EF) also improved during the study period following the same pattern. However, parameters of diastolic function, such as lateral e′, septal e′, E/e′ ratio, E/A ratio and LAVI did not change, suggesting that LV filling pressures were not significantly affected by the allocated treatments. We also observed a reduction in left ventricular wall thicknesses, and in LVMI and RWT measurements across the study period, regardless of the allocated treatment (Table 3).

The performance of indirect measurements of body fluid displacement using bioimpedance and neck circumference at both baseline and the end of the study showed no statistically significant differences between the intervention groups (Table 4).

Intra-observer reproducibility was estimated from a random subsample of 20 studies. Coefficients of variation (1.9%) and intraclass correlation coefficient (LVEF; ICC = 0.86 (95% CI = 0.64–0.95), as a proxy for bidimensional measurements, CV = 2.1% and ICC = 0.99 (95% CI = 0.97–0.99), as a proxy for Doppler measurements for E/e′; CV = 6.6% and ICC = 0.88 (95% CI: 0.78–0.98) for LV-GLS and CV = 7.5% and ICC = 0.65 (95% CI = 0.40–0.90)) for RV-GLS.

There were non-statistically-significant differences in self-reported adverse events between the two groups at the end of the study. However, it is worth noting that a slightly higher proportion of participants in the amlodipine group reported experiencing lower limb edema (48.4%) than that in the diuretic group (27.3%), although this disparity was not found to be statistically significant (*p* = 0.12).

## 4. Discussion

We demonstrated that treatment of high blood pressure for eight weeks in patients with OSA produced limited changes in echocardiographic metrics, with possible effects on left ventricular systolic function as measured via GLS and in LV geometry, but not on LV diastolic function parameters or on right ventricular function measured by GLS. There were no differences between diuretics (chlortalidone + amiloride) and amlodipine, despite the rationale suggesting the mechanistic benefit of diuretics in OSA patients.

Preliminary analysis from our data suggested that neither the administration of diuretics nor that of amlodipine had a treatment effect on the intensity of OSA suggesting that modifications in LV systolic function and LV structural parameters may be related to the blood pressure lowering of antihypertensive drugs rather than to the AHI reduction [14].

The movement of fluid from the lower limbs to the neck area during the night altering upper airway dynamics seems to be involved in the physiopathology [6] and severity [17] of OSA in hypertensive patients. Combining metolazone with spironolactone led to a noteworthy enhancement in the AHI in individuals with OSA and poorly controlled hypertension. This approach helped decrease the amount of fluid buildup in the neck region and larynx, per a study. Another trial with 15 participants reported similar outcomes through the application of intensive, short-term diuretic treatment with intravenous furosemide alongside spironolactone, further emphasizing the potential efficacy of this mechanism [8]. Another recent randomized clinical trial found that in men with severe OSA, diuretic therapy or a sodium-restricted diet resulted in a slight AHI decrease compared to that with a placebo [18].

Hypertension is likely to play a key role in LV subclinical dysfunction present in OSAS. There is robust evidence demonstrating that CPAP improves blood pressure control in patients with OSA and resistant hypertension [10]. It is also demonstrated in diverse population groups that antihypertensive treatment improves LV-GLS, especially among individuals who receive higher doses of antihypertensives and have subclinical systolic dysfunction [19,20]. Even in hypertensive individuals without signs of cardiac target organ disease, there are already subtle alterations in systolic function parameters [21], and blood pressure reduction is associated with favorable cardiac remodeling, but not with diastolic parameters [22]. These findings reflect those of our study, where participants had OSA and hypertension, but no evidence of target organ damage based on echocardiographic parameters.

The effects of CPAP on LV diastolic function have shown conflicting results in the literature [23,24,25,26,27,28], and we did not find significant effects of BP-lowering drugs on diastolic function parameters, suggesting a limited role for modifications of the volemic state or cardiac filling properties in participants without overt cardiovascular disease. The current evidence is also conflicting regarding the relationship between hypertension and RV systolic function impairment, which could be caused by increased RV filling pressures [29], which were not seen in our study. These findings suggest an alternative rationale following the direction that fluid accumulation and rostral shift might be of a lower magnitude than previously expected, and that a direct effect of antihypertensive drugs on LV function parameters and cardiac remodeling is likely to be involved in this group of patients.

Certain limitations should be considered when interpreting the results. The participants had a relatively low previous burden of hypertension, as indicated by their echocardiographic parameters falling within normal limits. This may have made them less susceptible to changes resulting from the intervention. The marginal significance of the treatment effect over time on echocardiographic parameters, regardless of the specific drug administered, may be secondary to the insufficient statistical power of our study.

## 5. Conclusions

The administration of diuretics or amlodipine had small and similar effects on echocardiographic parameters in patients with moderate OSA and hypertension. The possibility that blood pressure-lowering drugs have a differential effect in patients with OSA requires further investigation in studies with larger samples.

## Figures and Tables

**Table 1:** Baseline characteristics of the participants with obstructive sleep apnea and hypertension randomized to diuretics (chlorthalidone + amiloride) or amlodipine.

	Diuretics N = 27	Amlodipine N = 28
Male sex (%)	21 (77.8)	19 (67.9)
Age (years)	51.7 ± 6.9	54.9 ± 8.1
Body mass index (kg/m^2^)	29.4 ± 3.3	29.8 ± 4.6
Neck circumference (cm)	40.5 ± 4.5	40.9 ± 3.5
24 h SBP (mmHg)	135.5 ± 14.4	136.0 ± 13.3
24 h DBP (mmHg)	83.9 ± 7.5	84.0 ± 7.9
Apnea-hypopnea index (events/h)	33.4 ± 9.3	29.8 ± 9.8
Creatinine (mg/dL)	0.9 ± 0.2	0.9 ± 0.2
Potassium (mmol/L)	4.5 ± 0.3	4.5 ± 0.3
Total body water (kg)	45.3 ± 7.9	43.5 ± 7.8
NT-pro-BNP (pg/mL)	38.0 ± 30	38.0 ± 37

Data presented as mean ± SD or N (%).

**Table 2:** Twenty-four-hour ambulatory blood pressure monitoring (in mmHg) at baseline and 8 weeks after randomization to diuretics (chlorthalidone + amiloride) or amlodipine.

	Diuretics			Amlodipine			*p*-Value ^բ^
	Baseline	Follow-Up	*p*-Value ^∞^	Baseline	Follow-Up	*p*-Value ^∞^	
24 h SBP	135.8 ± 13.9	124.9 ± 7.4	<0.001	136.6 ± 13.1	125.0 ± 8.8	<0.001	0.8
24 h DBP	83.7 ± 7.2	78.8 ± 5.1	<0.001	84.0 ± 7.6	78.7 ± 5.0	<0.001	0.8
Daytime SBP	140.4 ± 14.3	130.0 ± 7.9	<0.001	141.0 ± 12.4	128.8 ± 9.4	<0.001	0.6
Daytime DBP	86.6 ± 7.3	81.5 ± 6.0	<0.001	86.9 ± 7.5	81.0 ± 4.9	<0.001	0.6
Nighttime SBP	126.8 ± 16.8	113.7 ± 10.9	<0.001	127.2 ± 17.3	117.0 ± 11.6	0.008	0.5
Nighttime DBP	77.5 ± 7.7	72.7 ± 5.8	0.002	77.5 ± 9.4	73.6 ± 7.0	0.013	0.6
Nocturnal SBP dipping	16 (59.3)	20 (74.1)	0.16	14 (56.0)	10 (40.0)	0.3	0.01 ^λ^
Nocturnal DBP dipping	14 (51.9)	16 (59.3)	0.4	12 (48.0)	11 (44.0)	0.8	0.3 ^λ^
24 h Heart Rate	70.6 ± 8.0	73.5 ± 6.9	0.002	69.2 ± 7.7	72.4 ± 9.9	0.006	0.8

Data presented as mean ± SD or N (%); ^∞^ in-group paired *t*-test comparison; ^բ^ independent *t*-test for comparison in variation differences between groups; ^λ^ Pearson’s χ^2^ between groups. SBP: systolic blood pressure (mmHg); DBP: diastolic blood pressure (mmHg).

**Table 3:** Echocardiographic bidimensional (2D-echo), Doppler, and speckle tracking global longitudinal strain (GLS) parameters at baseline and 8 weeks after randomization to diuretics (chlorthalidone + amiloride) or amlodipine.

	Diuretics			Amlodipine			*p*-Value ^բ^
	Baseline	Follow-Up	*p*-Value ^∞^	Baseline	Follow-Up	*p*-Value ^∞^	
LVDD (mm)	47.2 ± 4.4	47.1 ± 4.9	0.87	47.2 ± 5.0	47.5 ± 4.8	0.58	0.63
IVST (mm)	10.2 ± 1.0	9.8 ± 1.1	0.04	10.7 ± 1.5	10.1 ± 2.0	0.09	0.6
PWT (mm)	9.3 ± 1.2	8.7 ± 1.4	0.001	9.5 ± 1.3	8.6 ± 1.2	<0.001	0.36
LVMI (g/m^2^)	80.7 ± 17.7	74.6 ± 16.2	0.009	85.6 ± 18.4	78.4 ± 20.0	<0.001	0.72
RWT	0.40 ± 0.05	0.37 ± 0.07	0.002	0.41 ± 0.06	0.37 ± 0.06	0.01	0.31
EF(%)	63.6 ± 5.1	65.7 ± 5.4	0.03	63.9 ± 5.4	66.6 ± 4.6	0.03	0.8
LAVI (mL/m^2^)	29.4 ± 9.6	28.5 ± 8.7	0.38	31.3 ± 6.6	30.9 ± 7.6	0.71	0.69
LVOT-VTI (cm)	19.6 ± 3.1	19.2 ± 3.7	0.46	20.3 ± 3.2	20.5 ± 3.7	0.70	0.21
E-wave (cm/s)	67.6 ± 12.9	67.4 ± 13.7	0.90	69.8 ± 14.4	70.4 ± 12.8	0.80	0.79
A-wave (cm/s)	65.9 ± 17.6	62.2 ± 16.0	0.09	66.7 ± 19.0	67.7 ± 19.7	0.68	0.15
E/A ratio	1.11 ± 0.44	1.12 ± 0.29	0.73	1.11 ± 0.34	1.10 ± 0.31	0.71	0.62
Lateral e′ (cm/s)	11.6 ± 2.7	11.9 ± 2.7	0.33	11.0 ± 2.4	10.5 ± 2.5	0.21	0.12
Septal e′ (cm/s)	7.6 ± 1.8	7.6 ± 1.5	1.0	7.5 ± 1.9	7.5 ± 1.5	0.86	0.92
Mean E/e′ ratio	7.26 ± 1.64	7.07 ± 1.62	0.39	7.77 ± 1.89	8.1 ± 1.94	0.35	0.21
LV-GLS (%)	−22.3 ± 3.4	−23.5 ± 3.4	0.034	−21.5 ± 3.4	−22.8 ± 3.5	0.048	0.90
RV-GLS (%)	−25.4 ±5.2	−25.2 ±3.8	0.85	−24.2 ±3.7	−24.4 ±3.6	0.84	0.79

Data presented as mean ± SD; ^∞^ in-group paired *t*-test comparison; ^բ^ independent *t*-test for comparison of variation differences between groups; LVDD: left ventricular diastolic diameter; IVST: interventricular septal thickness; PWT: posterior wall thickness; LVMI: left ventricular mass index; RWT: relative wall thickness; EF: biplanar Simpson’s ejection fraction; LAVI: biplanar left atrial volume index; LVOT-VTI: left ventricular outflow tract velocity = time integral; E-wave: early mitral peak velocity; A-wave: late (atrial) mitral peak velocity; lateral e′: lateral mitral annulus displacement peak velocity; septal e′: septal mitral annulus displacement peak velocity; LV-GLS: left ventricular global longitudinal strain; RV-GLS: right ventricular global longitudinal strain.

**Table 4:** Indirect measurements of body fluid displacement at baseline and 8 weeks after randomization to diuretics (chlorthalidone + amiloride) or amlodipine.

	Diuretics			Amlodipine			*p*-Value *
	Baseline	Follow-Up	*p*-Value ^∞^	Baseline	Follow-Up	*p*-Value ^∞^	
Total body water	44.4 ± 9.3	43.6 ± 9.3	0.8	44.3 ± 8.0	43.8 ± 8.0	0.9	0.8
Neck circumference	39.6 ± 4.9	41.1 ± 4.8	0.3	41.1 ± 3.4	41.1 ± 3.7	0.3	0.5

* Two-way ANOVA for treatment and time; **^∞^** *p* for interaction.

## Data Availability

The data presented in this study are available on request from the corresponding author. The data are not publicly available due to ethical reason.
